# Enhancing Food Safety and Infection Control in Mass Foodservice Operations: Implementing a Foodservice Provision Audit Tool for Sport (FPAT‐S)

**DOI:** 10.1111/jhn.70117

**Published:** 2025-09-01

**Authors:** Angela C. Dufour, Fiona E. Pelly, Hattie H. Wright, Rachael Thurecht

**Affiliations:** ^1^ School of Health University of the Sunshine Coast Sunshine Coast Queensland Australia

**Keywords:** athlete, food safety, foodservice, illness, infection control, public health

## Abstract

Ensuring food safety and infection control in large‐scale foodservice operations is critical to protecting health in mass feeding environments, including athletes' dining halls and institutional dining settings. Athletes at major competitions are at risk of illness in overcrowded dining areas, especially where foodservice safety standards are inconsistent.

**Objectives:**

This study aimed to (1) develop and pilot‐test a Foodservice Provision Audit Tool for Sport (FPAT‐S) at two major sport competitions to evaluate compliance with infection control and food safety measures, and (2) determine its inter‐rater reliability.

**Methods:**

The FPAT‐S was tested by health professionals during the 2022 Canada Summer (*n* = 12) and 2023 Winter Games (*n* = 9). The tool included 19 questions with binary, multiple‐choice, and Likert scale responses. Compliance trends over time and inter‐rater reliability were analyzed.

**Results:**

Hand sanitizer availability exceeded 75% compliance and improved over time, whereas staff sanitation compliance declined by the end of both events. Physical distancing compliance remained below 50% throughout. Binary response questions showed moderate agreement (κ = 0.471, *p* = 0.028) between auditors compared to scale and multiple‐choice questions during the Summer phase, while agreement was lower and non‐significant for scale and multiple‐choice responses in both phases. Auditor variability was attributed to subjectivity and audit timing.

**Conclusion:**

The FPAT‐S provides a structured approach to assessing food safety and infection control in mass foodservice settings. With refinement, it can support dietitians and foodservice managers in maintaining compliance across a range of institutional and commercial operations, beyond sporting events, and inform future public health infection control strategies.

## Summary

- A new audit tool: Foodservice Provision Audit Tool for Sport (FPAT‐S) was developed and tested to assess food safety and infection control in foodservice sport operations.
- Tool results revealed strong compliance for hand sanitizer availability, but variable compliance for staff sanitation and physical distancing measures to control infection transmission.
- FPAT‐S binary response items were most reliable for consistent auditing across raters.
- The FPAT‐S offers a structured, practical method for dietitians and foodservice managers to monitor and improve compliance with food safety and infection control measures in large‐scale dining settings.

## 1 Introduction

Mass foodservice operations, including those in healthcare, institutional dining, and major sporting events, must adhere to stringent food safety and infection control measures to protect public health. Large‐scale dining environments, such as those at major sporting events, hospitals, and university cafeterias, present unique challenges due to high food production volumes, rapid meal service, and shared dining spaces. Major sporting events, such as Canada Games (www.canadagames.ca), gather a large number of athletes and staff for competition, camaraderie, and cultural exchange. Ensuring athlete health is crucial at these events, as acute illness, often attributed to foodborne pathogens, poses a significant threat, potentially affecting performance and the integrity of the games [1, 2, 3]. Although evidence of disease transmission at international sports mass gatherings is unclear, the inclusion of large‐scale foodservice operations in enclosed indoor spaces, like dining halls, poses a high risk for spreading infectious diseases [4]. Historically, studies on foodservice environments have focused on foodborne illnesses from bacteria, while viruses have received comparatively less attention [5].

Concerns about food safety at international sporting events have persisted over the last two decades, with better hygiene measures encouraged [6]. At the Sydney 2000 Olympic Games, food inspections revealed non‐compliance with food safety standards and other previous games highlighted poor hygiene practices like improper handwashing and hazardous food storage [7, 8]. Athlete illness rates at subsequent Olympic Games have ranged from 5% to 9%, with gastrointestinal and respiratory illnesses being most common [9, 10]. Poor nutrition labelling and inconsistent caterer compliance have been cited as contributing factors to these illnesses [11, 12].

Food safety concerns also arose during the Beijing 2008 Olympics, where local meat contamination with clenbuterol raised fears of inadvertent doping violations. Despite forming a food safety subcommittee, which utilized the Hazardous Analysis Critical Control Points (HACCP) principles and guidelines to address such issues, food safety continued to be inadequate [13, 14, 15]. Food safety risks at events vary based on location and caterer compliance, as seen during the PyeongChang 2018 Winter Olympics, where hygiene concerns persisted [11].

In response to the COVID‐19 pandemic, infection control measures, such as enhanced sanitation, physical distancing, and modifications to foodservice delivery, were implemented at events like the Olympic and Paralympic Games [16, 17]. Despite these countermeasures showing promise in reducing illness at events [10], continuity in countermeasures included in foodservice guidelines, especially post‐pandemic, appears to be lacking. Most guidelines from organizations like the World Health Organization (WHO) and the Food and Drug Administration (FDA) have not been updated since 2020–2021 [18, 19], and thus emphasize that these guidelines would still be appliciable in a post‐pandemic environment. The WHO continues to advocate for physical distancing, mask‐wearing, and stringent hygiene practices, such as use of disposable products, prohibition of buffet‐style service, reduced seating times and inclusion of sneezeguards. All of these are suggested by the WHO to be implemented to minimize disease spread at mass gatherings, including sporting events [20]. The FDA suggests that frequent cleaning and sanitation of commonly touched surfaces in foodservice environments is preferred [21]. The WHO's Mass Gathering Risk Assessment Tool for Sports evaluates the risk of COVID‐19 transmission but does not offer specific guidelines for safe food practices [22]. There continues to be an ongoing need to ensure food safety, however, the implementation and evaluation of new and effective infection control measures are challenged by caterers in this setting. In a recent study, food service stakeholders (caterers, organizers) perceived that the development of other foodservice standards would increase their ability to better comply with any new foodservice and safety guidelines [23].

Traditional food safety audits at major sporting events focus on back‐of‐house areas, such as microbiological testing and monitoring food holding temperatures, usually as part of standard HACCP monitoring programs [7, 24]. External audit tools have been previously used for general menu suitability at major sporting events [8, 25, 26, 27] or for food safety compliance, such as the Safe Quality Food (SQF) Code at the Tokyo 2020 Olympics. At the Canada Games, caterers were required to adhere to Safe Food for Canadians Regulations, which include HACCP principles, but no public audit data verifies compliance at these events [28]. Currently, there is no standardized and validated audit tool that focuses on front‐of‐house foodservice provisions in mass feeding environments, such as athlete dining halls, to assess compliance with food safety and infection control measures. Such a tool would provide objective evidence of safety in dining environments, reducing the risk of disease transmission and ensuring compliance with infection control guidelines.

This study aimed to develop and pilot test a Foodservice Provision Audit Tool for Sport (FPAT‐S) in mass feeding environments, with an initial focus on athlete dining halls at two major sporting events. The objectives were to evaluate compliance with food safety and infection control measures, including those introduced during COVID‐19, and to determine if compliance varied over the course of the games. Additionally, the study aimed to assess the tool's inter‐rater reliability to ensure consistent application by multiple auditors, reducing subjective bias and variability in evaluations. The findings from this study have implications for dietitians, foodservice managers, and public health professionals working in various mass foodservice operations, including healthcare, institutional, and commercial settings.

## 2 Methods

A variety of information sources were considered to identify quality indicators (audit items) to inform the structure of the FPAT‐S. The tool was formatted as a checklist with binary, Likert scale and multiple‐choice questions to be used by the auditor to record visual observations in the mass feeding environment. The audit process was adapted from established methods used in the foodservice industry, incorporating foodservice policies and safety measures specific to mass feeding in major sporting event environments [16, 17, 21, 29]. Literature informing the audit items targeted behaviours that were relevant to food safety and infection control measures in the mass feeding environment, and it included food safety measures such as appropriate food holding temperatures [30] and infection control measures such as staff and patron personal hygiene, sanitation, and physical distancing [16, 17].

Audit items were included in the FPAT‐S based on their relevance to at least one target behaviour, being directly observable, and measured or determined objectively where feasible [31]. While some conditions could be measured objectively, other questions could, in practical terms, be measured and interpreted subjectively. For this reason, open‐ended responses were included to understand better participant perspectives and any factors influencing their responses. Ethics were approved for this study by the Human Research Ethics Committee of the University of the Sunshine Coast, S211540.

### 2.1 Audit Tool Development

To establish content and face validity, the researchers, who were experienced in foodservice safety within athlete feeding environments, drafted and reviewed the audit tool. They assessed whether the tool was appropriate, relevant, and suitable for evaluating foodservice safety and infection control provisions in the athlete setting and be able to be completed by non‐experienced foodservice professionals. This included a systematic content analysis to assess the comprehensiveness, relevance, and clarity of the audit items, as well as to ensure that the tool effectively captured key food safety and COVID‐19 infection control measures. Identification and categorization of audit items within the tool were each classified based on the specific aspect of food safety or COVID‐19 infection control they addressed (e.g., temperature control, sanitation practices, physical distancing). Each of the expert researchers independently reviewed each audit item for relevance, clarity, and appropriateness, and any missing items, until consensus was reached.

Audit tool questions were then organized into three sections: (1) Foodservice safety standards and COVID‐19 countermeasures; (2) Menu/allergen and directional signage for physical distancing; and 3. General mealtime observation as outlined in the ‘Minimum Operating Standards for Food Service at Canada Games’ [32]. The final FPAT‐S questionnaire consisted of 19 total questions with optional open‐ended responses for each, to allow participants to add observations, where relevant. The final question was included to provide an indication of how busy the dining hall was at the time of the audit, to allow for a better context of auditor responses.

### 2.2 Audit Tool Administration

The FPAT‐S was administered in the unique context of high‐volume dining halls at major multi‐sport events, where athletes and staff were served thousands of meals daily within tight operational windows. Auditors conducted real‐time, in‐person observations during peak mealtimes to capture dynamic foodservice operations, including food handling practices, sanitation procedures, and physical distancing. The audits were conducted independently and discreetly, without interfering with foodservice staff, using hard copy forms to record visible compliance or non‐compliance with each audit item. This approach aimed to reflect authentic conditions and supported the identification of both objective and subjective responses, which may have been influenced by environmental variability, interpretation of visual cues, or the complexity of the setting.

### 2.3 Audit Tool Pilot Test

The FPAT‐S was tested in two separate phases: Phase 1: 2022 Canada Summer Games in Niagara, Ontario (August 2022; 5000 participants and 18 sports) and Phase 2: 2023 Canada Winter Games in Charlottetown, Prince Edward Island (February–March 2023; 3600 participants and 20 sports) (www.canadagames.ca). Health and sports science professionals were purposively invited to participate on multiple occasions, based on their attendance during the Canada Summer and Winter Games and selected due to convenience and availability at the games.

Audits were distributed via email, and instructions informed auditors to print multiple copies and perform the audit in hard copy on multiple occasions as needed. Audits were then collected either via scanned copies by email to the principal researcher or by postal mail.

To assess the consistency of the audit tool between two independent auditors in each phase, inter‐rater reliability was measured using proportion analysis and Cohen's Kappa statistical analysis with IBM SPSS Statistics (Version 27) [33]. This statistical method was chosen because it accounts for the possibility of agreement occurring by chance between two auditors, making it a more robust measure than simple percentage agreement. Kappa values are interpreted as: values ≤ 0 = no agreement, 0.01–0.20 = none to slight, 0.21–0.40 = fair, 0.41–0.60 = moderate, 0.61–0.80 = substantial and 0.81–1.00 = almost perfect agreement [34]. Results of each same time audit were then categorized into responses that were the same or different between auditors and organized based on the response type of the question (binary yes/no (*n* = 9), multiple choice (*n* = 2) or scale (*n* = 8)).

Open comments were broadly coded into five categories around physical distancing, food safety, sanitization and cleaning, communication and signage, and dining hall description by countermeasure. Categories were originally organized based on countermeasure comments by (AD). The research team interpreted the meaning of auditor responses through an iterative process until consensus was reached. Interpretations of audit questions were determined based on their relevance to response type to help explain any variability between same‐time auditor responses or variances in experiences, perceptions and observations.

## 3 Results

### 3.1 Pilot Test of FPAT‐S to Determine Compliance to Foodservice Safety and Infection Control Measures

**Figure 1:**
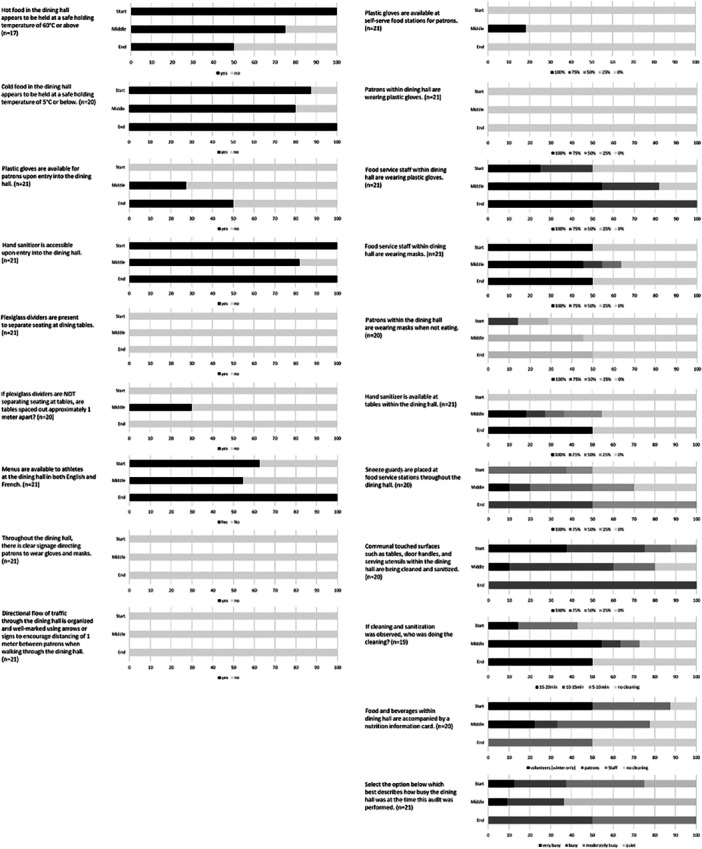
Combined compliance from Canada Summer and Winter Games of FPAT‐S questions by time of audit during Games (*n* = 21).

A total of 21 audits were conducted, which include 12 audits (3 auditors at the Canada Summer Games) and 9 audits (4 auditors at the Canada Winter Games) during various times throughout the games. Refer to Table S1. Combined results from both games revealed that food safety questions relating to food temperature showed 58% (*n* = 7) non‐compliance (‘no’ responses) to adequate hot holding temperatures and 75% (*n* = 9) non‐compliance to cold holding temperatures of food. There was less than 55% compliance in both Canada Summer and Winter games to physical distancing strategies such as; plexiglass dividers at dining tables, clear signage for mask and glove wearing, and directing traffic throughout the dining hall. There was also less than 55% compliance to sanitization strategies such as regular cleaning of communal surfaces, which was independent of the time the audit was taken during the games, refer to Figure 1. Results showed that there was over 80% compliance with hand sanitizer available at the entry into the dining halls, which was unaffected by the time of the audit during the games. There was, however, less than 50% compliance with sanitizers being made available at the dining tables, with 0% compliance at the start of games. There was less than 50% compliance for gloves available at entry, and less than 20% compliance with gloves being available at self‐service stations, throughout the duration of the games. Timing during the games (beginning, middle, end) did not affect compliance of the following audit items; signage directing patrons for mask and glove wearing, directing flow of traffic/distancing, patrons wearing plastic gloves, and plexiglass dividers on tables. These audit items were rated as non‐compliant throughout the games.

Audit items such as menu availability and plexiglass dividers at service stations appeared to have higher compliance at the end of games as compared to the beginning and middle.

Auditor comments revealed there were five themes around food safety and infection control that had subjective interpretation of their relevant audit questions: food safety (temperature control), distancing countermeasures, communication and signage, sanitization and cleaning and dining hall description (refer to Table 1). For example, comments relating to food safety regarding food holding temperature questions revealed that some auditors evaluated based on personal (subjective) observation, and others asked for temperature logs or witnessed actual temperatures. Comments related to communication and signage with respect to menu availability were interpreted subjectively by auditors on whether they were available either via hard copies, SmartApp or QR codes. Comments around sanitization and cleaning reflected that the auditors varied in their responses based on the areas specifically they were referring to at the time of the audit.

**Table 1:** Interpretation of auditor comments relevant to countermeasure audit question.

Code	Countermeasure	Interpretation	Example of auditor responses
Distancing countermeasures	Are tables spaced out 1 m apart	Indicates that responses were varied as distancing was dependent on the size/length of table	*Not every table, the long tables are at a 1 m of distance, small tables and high tables are not* *Depending on the table (long vs. short table)*
	Sneezeguards are placed at food service stations	Indicates variability based on the location auditors observed in the dining hall.	*Not at bread, fruits, hot food, in self serve foods* *None at hot buffet self serve stations* *Only 50% of the dining hall had sneeze guards in place (servery)* *In special diets, at fruit, not at toast, hot food or cereal*
Food safety	Food temperature – Hot foods	Indicates that the auditors were varied in how they determined if the food was hot: some asked for logs, some took temps, hot to touch, visibility of steam	*Asked foodservice staff and observed temperature log sheets* *Yes, meat have steam. No, veg & potatoes don't look hot* *No steam from hot foods, no cover on hot foods, pasta are not in a hot table* *Not verified* *Temperatures were taken while I was here*
	Food temperature – Cold foods	Indicates that the auditors were varied in how they determined if the food was cold: some asked for logs, some took temps, cold to touch, on ice	*Dishes cold to touch, ice around foods* *Foods look cold and the cold table is surrounded with ice, the dishes are cold to touch* *Asked foodservice staff and observed temperature log sheets* *Not verified*
Communication and signage	Menus are available	Indicates there was variability on how auditors responded based on when and how the menus were distributed, that is, QR code or printed version at stations	*QR code not working; no menu signage list at table* *Some are missing: yogurt, dessert, hot food* *Only available daily, cannot view other days*
Communication and signage	Food and beverages within dining hall are accompanied by a nutrition information card	Variability in responses based on whether auditors were reporting on allergen information or specific nutrition information on the label and on where/how they were displayed	*Not consistent on all items ‐ in the salad bar area* *Nutrition not available at muffins, danishes, etc. Nutrition information does not include calories or fiber* *No information on beverages. Carbs, protein, fat, sodium, allergens, ingredients* *Allergens on TV monitors with entree items, one signage with macro break down, @ point of service kcal/serving label*
	Throughout the dining hall, there is clear signage directing patrons to wear gloves and masks	Variable responses dependant on where specifically in the dining hall they were observing	Only a sign at the entry to wear a mask inside (no sign in the dining hall)
Sanitization/cleaning	Food service staff are wearing gloves/masks	Variable responses and subjective to the time the audit was taken (or whether observed over the entire meal period) and location within the dining hall	*A few were not – carrying plates of muffins, etc.*
	Hand sanitizers are available at tables	Variable response dependant on the location, specifically in the dining hall	*Only located on side tables (ex: condiments and grain area)* *At different areas in the dining hall*
	Communal touched surfaces such as tables, door handles, and serving utensils within the dining hall are being cleaned and sanitized.	Variable responses dependant on the specific area i.e responses indicated tables only.	*Tables were cleaned every 20 min*
Dining hall description	Which best describes how busy the dining hall was at the time this audit was performed.	Variability in responses based on the time when they started and ended the audit and where specifically in the dining hall they were referring to	*Became busy within 20 min after completing the audit (approx 100+ in line). 2nd servery opened to decrease* *Bottlenecking. +++ bottleneck by beverages, garbages, and dish pit around 6:15 PM*

### 3.2 Reliability of the Audit Tool

At the same time, audit responses had the same responses for 11/19 (0.58) and 10/19 (0.53) questions, respectively, and different responses for 8/19 and 9/19 audit questions, respectively, for the two phases (Summer and Winter). Refer to Table 2. Based on the response type (binary, scale or multiple choice), results showed that there was a greater consistency between rater responses in 6/9 (0.6) to binary response questions compared to 3/8 (0.38) and 1/2 for scale and multiple‐choice questions, respectively, at both games.

**Table 2:** Inter‐rater reliability comparing same time audit responses between two auditors at two separate events.

Contextual information for audits	Summer (*n* = 2)	Winter (*n* = 2)
How busy the dining hall was at the time this audit was performed.	Same^b^	Different^c^
Meal period	Breakfast service	Lunch service
Time of audits	6:50 AM and 7:55 AM	12:06 PM and 1:40 PM
Duration (min)	13 and 15 min	12 and 50 min
*FPAT‐S binary questions* ^a^		
*Food safety questions*		
1. Hot food in the dining hall appears to be held at a safe holding temperature of 60°C or above.	Missing data	Different
2. Cold food in the dining hall appears to be held at a safe holding temperature of 5°C or below.	Missing data	Same
*Infection control questions*		
3. Menus are available to athletes at the dining hall in both English and French.	Same	Same
4. Plexiglass dividers are present to separate seating at dining tables.	Same	Same
5. If plexiglass dividers are not separating seating at tables, are tables spaced out approximately 1 m apart?	Same	Different
6. Hand sanitizer is accessible upon entry into the dining hall.	Same	Different
7. Throughout the dining hall, there is clear signage directing patrons to wear gloves and masks.	Same	Different
8. Directional flow of traffic through the dining hall is organized and well‐marked using arrows or signs to encourage distancing of 1 m between patrons when walking through the dining hall.	Same	Same
9. Plastic gloves are available for patrons upon entry into the dining hall.	Different	Same
Level of agreement (proportion)	0.86	0.67
Kappa^d^	0.471	0.182
Significance (*p*‐value)	0.028	0.571
*FPAT‐S scale questions* ^a^		
1. Plastic gloves are available at self‐serve food stations for patrons.	Same	Different
2. Food service staff within dining hall are wearing plastic gloves.	Different	Same
3. Patrons within dining hall are wearing plastic gloves.	Same	Same
4. Patrons within the dining hall are wearing masks when not eating.	Different	Different
5. Hand sanitizer is available at tables within the dining hall.	Different	Different
6. Patrons within dining hall are wearing plastic gloves.	Same	Same
7. Food service staff within dining hall are wearing masks.	Same	Same
8. Food and beverages within dining hall are accompanied by a nutrition information card.	Missing data	Different
Level of Agreement (proportion)	0.57	0.50
Kappa^d^	0.097	0.222
Significance (*p*‐value)	0.694	0.254
*FPAT‐S multiple choice questions* ^a^		
1. Communal touched surfaces such as tables, door handles, and serving utensils within the dining hall are being cleaned and sanitized.	Different	Different
2. If cleaning and sanitization was observed, who was doing the cleaning	Same	Same
Level of agreement^d^	0.50	0.50
Kappa^d^	0.333	0.333
Significance (*p*‐value)	0.157	0.157
*Total agreement all questions*	0.69	0.53

^a^ Response type: Binary: Yes/No, Scale = 0, 25%, 50%, 75%, 100% for compliancy, multiple choice.

^b^ Same response between auditors.

^c^ Different response between auditors.

^d^ Questions with missing data were not included in kappa analysis.

Inter‐rater reliability on binary responses yielded a *k* = value of 0.471 (*p* = 0.028) and *k* = 0.182 (*p* = 0.571) for Summer and Winter Games, respectively, indicating a moderate level of agreement between the two Summer auditors and a none‐to‐slight level of agreement between Winter auditors. For scale responses, agreement between Summer auditors was none to slight and between Winter auditors was fair, however, both were not statistically significant (*k* = 0.097, *p* = 0.694 and *k* = 0.222, *p* = 0.254 for Summer and Winter, respectively). Multiple choice responses showed a fair level of agreement (*k* = 0.133) for both Summer and Winter phases, which were not statistically significant (*p* = 0.157).

## 4 Discussion

This study sought to develop and pilot‐test an FPAT‐S to assess food safety and infection control compliance in mass foodservice environments, initially applied in athlete dining halls at two major sporting events. The findings reveal significant non‐compliance with critical food safety standards, particularly regarding food temperature control, as well as infection control measures such as physical distancing and sanitation. These findings underscore the need for enhanced monitoring and intervention strategies applicable to a range of large‐scale foodservice settings, including hospitals, long‐term care facilities, and institutional dining operations. Non‐compliance with food temperature control may be partly due to differences in subjective interpretation of questions pertaining to food temperature control, which highlights the need for clearer guidelines to minimize subjective interpretation in future iterations of the tool.

Despite the heightened awareness of infection control measures following the COVID‐19 pandemic, this study found that compliance with physical distancing and sanitation measures remained low, suggesting challenges in integrating public health guidelines into large‐scale foodservice operations. Given the potential high risk for disease transmission in mass feeding environments, these failures in compliance are concerning and highlight the need for stronger public health interventions and evaluation.

Interestingly, compliance with certain infection control measures, such as hand sanitizer availability, showed improvement throughout the event, reaching over 80% compliance by the end of each event, possibly indicating that foodservice staff may not have had access to supplies or were too busy at the start of the games to comply. However, compliance with staff sanitation measures—including mask‐wearing, glove use, and cleaning communal surfaces—declined over time. This suggests that preventive measures were either not prioritized or inadequately reinforced as the games progressed. The lack of attention to preventable infections that can be controlled by the suggested countermeasures should be of high priority throughout the entire span of the event. Athletes and staff sometimes travel large distances and train for years to get to this stage to compete, thus maximizing their health at this pinnacle time in their careers, and this should be a continued focus.

Similar inconsistent food safety and infection control trends were reported at the Tokyo 2020 and Beijing 2022 Olympic and Paralympic Games, where inconsistencies in sanitization compliance were observed across multiple dining locations [35]. The availability of gloves and sanitizers at dining tables and self‐service stations consistently fell below 50%, potentially reflecting a sense of complacency or operational challenges in restocking supplies as the events progressed. Previous research suggests that foodservice staff compliance with safety measures is influenced by multiple factors, including workload, forgetfulness, inadequate training, and workplace culture [36]. These barriers highlight the importance of ongoing staff education and reinforcement strategies to sustain compliance. Given that public health risks persist beyond the pandemic, integrating infection control as a standard component of foodservice operations remains essential. Notably, compliance with physical distancing and signage directives did not improve, remaining consistently low throughout the games, suggesting that measures dictated by the physical environment are less controllable by foodservice staff. Recent studies indicate that the size and layout of dining facilities influence adherence to distancing protocols, with smaller, less crowded venues exhibiting higher compliance [35]. This highlights the health risks associated with poor food safety and infection control compliance in large‐scale sporting events. It reinforces the need for early collaboration between public health officials, foodservice managers, and event organizers to incorporate infection control considerations into venue design and operational planning with evolving safety guidelines [23]. As large‐scale foodservice operations transition to post‐pandemic protocols, maintaining a strong focus on infection control throughout the entire event duration remains critical.

Dietitians and foodservice managers play a key role in implementing and monitoring food safety and infection control measures in various settings, including hospitals, schools, and aged care facilities. This study underscores the need for standardized audit tools like FPAT‐S to assess compliance, identify areas for improvement, and ensure the safe provision of food in high‐risk environments.

A secondary aim of this study was to assess the inter‐rater reliability of the FPAT‐S to determine whether the tool provides consistent and accurate assessments of food safety and infection control compliance across different auditors in athlete mass feeding environments. This is the first time that this kind of tool was applied in this setting, and while the development incorporated review of the content, the reliability of the tool had not been tested previously. Applying the tool at a major sporting event, such as a national major game, provided an opportunity to test its reliability and application over the entire span of the games.

The results of the inter‐rater reliability analysis revealed varying levels of agreement between auditors, with binary response questions demonstrating greater consistency compared to scale and multiple‐choice questions. The higher reliability of binary questions may be due to their straightforward nature, while scale and multiple‐choice questions were more prone to subjective interpretation. However, Cohen's Kappa analysis indicated that some agreement observed in binary questions might be attributable to chance, warranting further refinement of response formats to enhance reliability.

Binary response questions in the Summer phase showed higher agreement compared to the Winter phase, though missing responses in the Summer phase may have influenced these findings. However, there is concern that a higher proportion of agreement ratings might be due to random chance because only two categories are examined [37]. For this reason, to quantitatively assess inter‐rater reliability, Cohen's Kappa statistic was calculated for binary, multiple‐choice and scale questions in each phase.

For scale response questions, agreement ranged from none to slight in the Summer phase and fair in the Winter phase, with neither reaching statistical significance. As Kappa values can be influenced by sample size, data distribution, and auditor bias, these findings suggest that while proportion agreement was moderate, variability in auditor interpretation remains a challenge. For these reasons, the kappa values may be lower here despite their higher values of proportion agreement [38].

The higher inter‐rater reliability observed with binary questions, particularly during the Summer phase, suggests that multiple‐choice and scale‐type questions may require refinement or additional auditor training to enhance reliability before future implementation. This will help to reduce random chance agreement among auditors. To improve reliability, future iterations of the tool should focus on refining questions with consistent response types and formats to ensure they are easily interpretable and minimize the chance of divergent interpretations among auditors [31]. Further reliability testing is also required upon final tool refinement. This is crucial from a public health surveillance standpoint, as variability between auditors can lead to inconsistent assessments of compliance, potentially underestimating or overestimating risks.

Results from the two‐phase test also allowed for interpretations on how the auditors responded to questions, so that further tool refinement can be made for application at higher risk events such as the Olympic and Paralympic Games. Auditor feedback indicated that discrepancies in responses could be attributed to differences in how questions were understood or the exact timing of audits. For example, inconsistencies in evaluating temperature compliance suggest that auditors employed different methods, such as visual inspection versus log verification. Implementing clear criteria—such as requiring direct temperature measurements—would reduce subjective interpretation and improve inter‐rater reliability. To reduce subjectivity and enhance the tool's accuracy, future revisions of the FPAT‐S should include clearer guidelines for auditors, as well as more objective, standardized observation methods [31]. This could involve providing a list of acceptable verification methods, such as direct temperature readings or inspection of logs.

Similarly, questions regarding menu and allergen signage may need clarification for auditors to ensure consistency in auditing, such as defining specifically what and how the information is displayed and where. While these measures may not directly impact the spread of communicable diseases, they are still important for preventing issues such as allergic reactions or gastrointestinal disturbances among athletes [6]. Future revisions should consider enhancing the clarity of these questions and incorporating new technologies, such as QR codes or mobile Apps, to facilitate menu and nutrition communication. The findings in this study show that a validated foodservice audit tool (such as FPAT‐S) can be used in public health monitoring systems, particularly in the context of food safety and infection control in mass feeding environments.

## 5 Conclusions

This study highlights the inconsistencies in food safety and infection control compliance in mass foodservice environments, reinforcing the need for structured monitoring tools and standardized guidelines among caterers and organizing committees. While some measures, such as menu availability and nutrition card provision, improved throughout the event, sanitation compliance declined, emphasizing the need for continuous enforcement of hygiene protocols.

The FPAT‐S shows promise as a standardized audit tool for evaluating compliance in large‐scale foodservice operations, but further refinement is necessary to enhance its reliability. Shifting towards binary response formats and strengthening auditor training will improve consistency in compliance assessments. With these modifications, the FPAT‐S can serve as a valuable tool for public health surveillance, supporting dietitians, foodservice managers, and policymakers in ensuring safe food provision in diverse mass feeding environments. By informing catering operations, event organizers, and public health agencies, the tool can contribute to improved compliance with food safety and infection control measures across institutional and commercial foodservice settings.

## Author Contributions

Fiona E. Pelly and Angela C. Dufour conceptualized the study methodology and design of the survey, with input from Hattie H. Wright. Fiona E. Pelly and Angela C. Dufour oversaw the data analysis, and all authors contributed to the interpretation of the data. Angela C. Dufour drafted the manuscript, and Fiona E. Pelly, Hattie H. Wright and Rachael Thurecht provided intellectual input to all sections of the paper. Angela C. Dufour, Fiona E. Pelly, Hattie H. Wright and Rachael Thurecht read and approved the final version of the paper.

## Ethics Statement

Ethics were approved for this study by the University of the Sunshine Coast, Human Research Ethics Committee, # S211540.

## Conflicts of Interest

Angela C. Dufour was employed by the Canadian Olympic Committee at the time the research was conducted. The other authors declare no conflicts of interest.

## 1 Peer Review

The peer review history for this article is available at https://www.webofscience.com/api/gateway/wos/peer-review/10.1111/jhn.70117.

## Supporting information


**Supplementary material Table 1:** Auditor responses at two separate events.

## Data Availability

The data that support the findings of this study are available in the supporting material of this article.
