# Supplementary material for: Enhancing Food Safety and Infection Control in Mass Foodservice Operations: Implementing a Foodservice Provision Audit Tool for Sport (FPAT‐S)
Source: J Hum Nutr Diet. 2025 Sep 1;38(5):e70117. doi: 10.1111/jhn.70117 (PMC12402678; doi:10.1111/jhn.70117)
Supplement: Supplementary file 1 — Supplementary material Table 1: Auditor responses at two separate events. [file JHN-38-0-s001.docx]

Supplementary material; Table 1 Auditor responses at two separate events

| **FPAT-S Questions** | **Game location** | | **Time frame during games** | | |
| --- | --- | --- | --- | --- | --- |
|  | **Summer** | **Winter** | **Start** | **Middle** | **End** |
|  | **n (%)** | **n (%)** | **n (%)** | **n (%)** | **n (%)** |
| **Q1: Hot food in the dining hall appears to be held at a safe holding temperature of 60°C or above (n=17)** |  |  |  |  |  |
| no | 2 (22.2) | 1 (12.5) | 0 (.0) | 2 (25.0) | 1 (50.0) |
| yes | 7 (77.8) | 7 (87.5) | 7 (100.0) | 6 (75.0) | 1 (50.0) |
| **Q2: Cold food in the dining hall appears to be held at a safe holding temperature of 5°C or below. (n=20)** |  |  |  |  |  |
| no | 2 (18.2) | 1 (11.1) | 1 (12.5) | 2 (20.0) | 0 (.0) |
| yes | 9 (81.8) | 8 (88.9) | 7 (87.5) | 8 (80.0) | 2 (100.0) |
| **Q3: Plastic gloves are available for patrons upon entry into the dining hall. (n=21)** |  |  |  |  |  |
| no | 8 (66.7) | 9 (100.0) | 8 (100.0) | 8 (72.7) | 1 (50.0) |
| yes | 4 (33.3) | 0 (.0) | 0 (.0) | 3 (27.3) | 1 (50.0) |
| **Q4: Plastic gloves are available at self-serve food stations for patrons. (n=21)** |  |  |  |  |  |
| 0% | 12 (100.0) | 7 (77.8) | 8 (100.0) | 9 (81.8) | 2 (100.0) |
| 25% | 0 (.0) | 0 (.0) | 0 (.0) | 0 (.0) | 0 (.0) |
| 50% | 0 (.0) | 0 (.0) | 0 (.0) | 0 (.0) | 0 (.0) |
| 75% | 0 (.0) | 0 (.0) | 0 (.0) | 0 (.0) | 0 (.0) |
| 100% | 0 (.0) | 2 (22.2) | 0 (.0) | 2 (18.2) | 0 (.0) |
| **Q5: Patrons within dining hall are wearing plastic gloves. (n=21)** |  |  |  |  |  |
| 0% | 12 (100.0) | 9 (100.0) | 8 (100.0) | 11 (100.0) | 2 (100.0) |
| 25% | 0 (.0) | 0 (.0) | 0 (.0) | 0 (.0) | 0 (.0) |
| 50% | 0 (.0) | 0 (.0) | 0 (.0) | 0 (.0) | 0 (.0) |
| 75% | 0 (.0) | 0 (.0) | 0 (.0) | 0 (.0) | 0 (.0) |
| 100% | 0 (.0) | 0 (.0) | 0 (.0) | 0 (.0) | 0 (.0) |
| **Q6: Food service staff within dining hall are wearing plastic gloves. ( n=21)** |  |  |  |  |  |
| 0% | 6 (50.0) | 0 (.0) | 4 (50.0) | 2 (18.2) | 0 (.0) |
| 25% | 0 (.0) | 0 (.0) | 0 (.0) | 0 (.0) | 0 (.0) |
| 50% | 0 (.0) | 0 (.0) | 0 (.0) | 0 (.0) | 0 (.0) |
| 75% | 2 (16.7) | 4 (44.4) | 2 (25.0) | 3 (27.3) | 1 (50.0) |
| 100% | 4 (33.3) | 5 (55.6) | 2 (25.0) | 6 (54.5) | 1 (50.0) |
| **Q7: Food service staff within dining hall are wearing masks. (n=21)** |  |  |  |  |  |
| 0% | 0 (.0) | 9 (100.0) | 4 (50.0) | 4 (36.4) | 1 (50.0) |
| 25% | 0 (.0) | 0 (.0) | 0 (.0) | 0 (.0) | 0 (.0) |
| 50% | 1 (8.3) | 0 (.0) | 0 (.0) | 1 (9.1) | 0 (.0) |
| 75% | 1 (8.3) | 0 (.0) | 0 (.0) | 1 (9.1) | 0 (.0) |
| 100% | 10 (83.3) | 0 (.0) | 4 (50.0) | 5 (45.5) | 1 (50.0) |
| **Q8: Patrons within the dining hall are wearing masks when not eating. (n=20)** |  |  |  |  |  |
| 0% | 6 (50.0) | 6 (75.0) | 5 (71.4) | 6 (54.5) | 1 (50.0) |
| 25% | 5 (41.7) | 2 (25.0) | 1 (14.3) | 5 (45.5) | 1 (50.0) |
| 50% | 0 (.0) | 0 (.0) | 0 (.0) | 0 (.0) | 0 (.0) |
| 75% | 1 (8.3) | 0 (.0) | 1 (14.3) | 0 (.0) | 0 (.0) |
| 100% | 0 (.0) | 0 (.0) | 0 (.0) | 0 (.0) | 0 (.0) |
| **Q9: Hand sanitizer is accessible upon entry into the dining hall. (n=21)** |  |  |  |  |  |
| no | 0 (.0) | 2 (22.2) | 0 (.0) | 2 (18.2) | 0 (.0) |
| yes | 12 (100.0) | 7 (77.8) | 8 (100.0) | 9 (81.8) | 2 (100.0) |
| **Q10: Hand sanitizer is available at tables within the dining hall. (n=21)** |  |  |  |  |  |
| 0% | 7 (58.3) | 7 (77.8) | 8 (100.0) | 5 (45.5) | 1 (50.0) |
| 25% | 0 (.0) | 2 (22.2) | 0 (.0) | 2 (18.2) | 0 (.0) |
| 50% | 1 (8.3) | 0 (.0) | 0 (.0) | 1 (9.1) | 0 (.0) |
| 75% | 1 (8.3) | 0 (.0) | 0 (.0) | 1 (9.1) | 0 (.0) |
| 100% | 3 (25.0) | 0 (.0) | 0 (.0) | 2 (18.2) | 1 (50.0) |
| **Q11a: Plexiglass dividers are present to separate seating at dining tables. (n=21)** |  |  |  |  |  |
| no | 12 (100.0) | 9 (100.0) | 8 (100.0) | 11 (100.0) | 2 (100.0) |
| yes | 0 (.0) | 0 (.0) | 0 (.0) | 0 (.0) | 0 (.0) |
| **Q11b: If plexiglass dividers are NOT separating seating at tables, are tables spaced out approximately 1 meter apart? (n=20)** |  |  |  |  |  |
| no | 10 (90.9) | 7 (77.8) | 8 (100.0) | 7 (70.0) | 2 (100.0) |
| yes | 1 (9.1) | 2 (22.2) | 0 (.0) | 3 (30.0) | 0 (.0) |
| **Q12: Sneeze guards are placed at food service stations throughout the dining hall. (n=20)** |  |  |  |  |  |
| 0% | 7 (63.6) | 0 (.0) | 4 (50.0) | 3 (30.0) | 0 (.0) |
| 25% | 0 (.0) | 4 (44.4) | 1 (12.5) | 2 (20.0) | 1 (50.0) |
| 50% | 1 (9.1) | 5 (55.6) | 3 (37.5) | 3 (30.0) | 0 (.0) |
| 75% | 2 (18.2) | 0 (.0) | 0 (.0) | 1 (10.0) | 1 (50.0) |
| 100% | 1 (9.1) | 0 (.0) | 0 (.0) | 1 (10.0) | 0 (.0) |
| **Q13a: Communal touched surfaces such as tables, door handles, and serving utensils within the dining hall are being cleaned and sanitized. (n=20)** |  |  |  |  |  |
| no cleaning | 6 (50.0) | 2 (25.0) | 4 (57.1) | 3 (27.3) | 1 (50.0) |
| 5-10 min | 0 (.0) | 3 (37.5) | 2 (28.6) | 1 (9.1) | 0 (.0) |
| 10-15min | 1 (8.3) | 0 (.0) | 0 (.0) | 1 (9.1) | 0 (.0) |
| 15-20min | 5 (41.7) | 3 (37.5) | 1 (14.3) | 6 (54.5) | 1 (50.0) |
| **Q13b: If cleaning and sanitization was observed, who was doing the cleaning? (n=19)** |  |  |  |  |  |
| no cleaning | 4 (33.3) | 0 (.0) | 1 (12.5) | 2 (22.2) | 1 (50.0) |
| staff | 7 (58.3) | 1 (14.3) | 3 (37.5) | 4 (44.4) | 1 (50.0) |
| patrons | 1 (8.3) | 0 (.0) | 0 (.0) | 1 (11.1) | 0 (.0) |
| volunteers (winter only) | 0 (.0) | 6 (85.7) | 4 (50.0) | 2 (22.2) | 0 (.0) |
| **Q14: Menus are available to athletes at the dining hall in both English and French. (n=21)** |  |  |  |  |  |
| no | 2 (16.7) | 6 (66.7) | 3 (37.5) | 5 (45.5) | 0 (.0) |
| yes | 10 (83.3) | 3 (33.3) | 5 (62.5) | 6 (54.5) | 2 (100.0) |
| **Q15: Throughout the dining hall, there is clear signage directing patrons to wear gloves and masks. (n=21)** |  |  |  |  |  |
| no | 12 (100.0) | 9 (100.0) | 8 (100.0) | 11 (100.0) | 2 (100.0) |
| yes | 0 (.0) | 0 (.0) | 0 (.0) | 0 (.0) | 0 (.0) |
| **Q16: Food and beverages within dining hall are accompanied by a nutrition information card. (n=20)** |  |  |  |  |  |
| 0% | 2 (18.2) | 0 (.0) | 0 (.0) | 2 (20.0) | 0 (.0) |
| 25% | 0 (.0) | 1 (11.1) | 1 (12.5) | 0 (.0) | 0 (.0) |
| 50% | 2 (18.2) | 1 (11.1) | 1 (12.5) | 2 (20.0) | 0 (.0) |
| 75% | 4 (36.4) | 6 (66.7) | 3 (37.5) | 5 (50.0) | 2 (100.0) |
| 100% | 3 (27.3) | 1 (11.1) | 3 (37.5) | 1 (10.0) | 0 (.0) |
| **Q17: Directional flow of traffic through the dining hall is organized and well-marked using arrows or signs to encourage distancing of 1 meter between patrons when walking through the dining hall. (n=21)** |  |  |  |  |  |
| no | 12 (100.0) | 9 (100.0) | 8 (100.0) | 11 (100.0) | 2 (100.0) |
| yes | 0 (.0) | 0 (.0) | 0 (.0) | 0 (.0) | 0 (.0) |
| **Q18: Select the option below which best describes how busy the dining hall was at the time this audit was performed. (n=21)** |  |  |  |  |  |
| quiet | 6 (50.0) | 3 (33.3) | 2 (25.0) | 7 (63.6) | 0 (.0) |
| moderately busy | 1 (8.3) | 3 (33.3) | 3 (37.5) | 0 (.0) | 1 (50.0) |
| busy | 3 (25.0) | 3 (33.3) | 2 (25.0) | 3 (27.3) | 1 (50.0) |
| very busy | 2 (16.7) | 0 (.0) | 1 (12.5) | 1 (9.1) | 0 (.0) |
